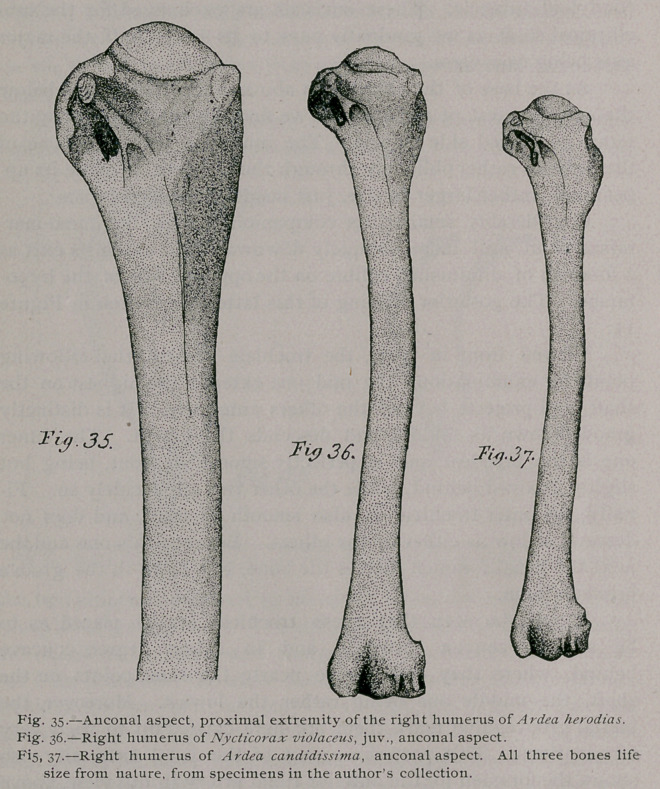# Osteological Studies of the Subfamily Ardeinæ

**Published:** 1889-10

**Authors:** R. W. Shufeldt


### THE JOURNAL

— OF —

### COMPARATIVE MEDICINE AND SURGERY.

VOL. X. OCTOBER 1889. NO. 4.

### ORIGINAL COMMUNICATIONS.

### ART. XVIII.—OSTEOLOGICAL STUDIES OF THE SUB-FAMILY ARDEINÆ.

BY R. W. SHUFELDT, M.D., C.M.Z.S.

[PART II.]

*Of the Pectoral Arch* :—Comparatively speaking the coracoid of the Great Blue Heron, is a large bone. Its sternal extremity is much spread out and quite thin and plate-like. Articular surfaces occur on both aspects of this end of the bone, for the fellow of the opposite side, and the sternum. One would think, and naturally, that these extremities of the coracoid would be quite unlike, from the fact that they cross each other in articulation, and are fitted in differently directed grooves on the sternum. Such, however, is not the case, for with the sole difference of a slight asymmetry of the articular facets, these bones are no more unlike than we find them in the majority of birds.

The shaft of a coracoid is slender and somewhat laterally compressed, a compression that is extended to the head of the bone, where it becomes decidedly marked. The summit of the bone being capped with a tuberous crown which curls over mesiad, and extends backwards to merge into the glenoid cavity. This latter is ample and fully two-thirds of the surface is afforded by the coracoid. The scapula process with the line of its articular surface at right angles to the long axes of both bones, is no larger than is just necessary to accommodate the head of the scapula. It never meets the furcula in any of the Herons that I have seen, and in all of these birds the bones of the pectoral arch are completely non-pneumatic.

Fig. 9.—Lower portion is an anterior view of the sternum of *Ardea herodias*, showing the decussation of the coracoidal grooves. Above, lifted from their position, are the coracoids with dotted line showing the extent to which they decussate. The scapulas are articulated above these, but the forchette has been removed. Same specimen as before. Life size from nature.

The coracoid of *A. candidissima* differs in no particular from the bone I have just described for *Ardea herodias;* while though *Nycticorax* also agrees in this respect with these birds in the main, it differs in having the inner angle of the expanded sternal end of the *right* coracoid truncate, instead of being drawn out into a point as the fellow of the opposite side is. This is due to the fact that the groove on the sternum has that shape in the Yellow-Crowned Night Heron.

The *scapula* among the *Ardeinæ*, generally, is a long narrow bone, with but a slight curvature from head to distal extremity. This latter is simply rounded off in *A. herodias* and in the Snowy Heron, but inclined to be slightly truncate in

*Nycticorax*. In the Great Blue Heron the head of the scapula is compressed from above, downwards, and much expanded in a transverse direction. Mesially it curls up a little to preserve the contour of the "tendinal canal," while on the opposite side, it supports an oblique, elliptical articular facet, constituting one-third of the glenoid cavity.

Among the Herons the furcula, or the united clavicles, is a very interesting bone in one or two particulars.

In figures 11 and 12 I present two views of this part of the pectoral arch, taken from a specimen in my own collection of *A. herodias*, it being the same individual from which all the drawings were made which illustrate this form. I would do this, even if a hundred skeletons of the same species were at my command, as it is better in many respects. One of the chief reasons is that each skeleton, even among birds, has its own individuality and ought to furnish all the figures if possible in any type monographed. The head of the *clavicle* in this Heron is tuberous, rather thickened, and evenly rounded off at its end. When articulated with the other bones of the arch, its superior border, quite close to this extremity, rests against the under side of the projecting summit of the mesial aspect of the coracoid. The rounded end of the furcula, from this point, reaches back a sufficient distance to barely escape touching the mesial and up-curled side of the scapular head, thus to all intents closing the tendinal canal by long walls ; its complete closure is really effected by the short ligament that holds these two bones *in situ* at this, their nearest point of approach. In some birds, as for instance certain diurnal Raptores, the canal is closed by the head of the furcula reaching the tip of the clavicular process of the coracoid. From the head of the bone to the hypocleidium a gradual reduction in size takes place, while the lateral compression is sustained throughout, at any rate until within a short distance from the latter part.

Now the hypocleidium of the clavicles in *Ardea herodias*, as in other herons, consists of both an inferior and a superior process (Figs. 11 and 12), both being in the same line. In our present subject the upper one is the larger of the two, while their common surface anteriorly is smooth and flat. Behind, it is rounded and marked by a longitudinal raised line. This latter feature in *Nycticorax* is raised to the rank of a well developed crest, and the lower process, in this bird, equals the upper in length, and as a whole is comparatively slenderer (Fig. 33).

Figure 34 gives a three-quartering view of the *furcula* of my specimen of *A. candidissima*. It will be observed that it differs in no important particular from *Ardea*, though the anterior surface of the lower process of the hypocleidium is longitudinally grooved, a feature which, by the way, I neglected to say, is faintly indicated in the latter heron.

A glance at any of the figures representing this bone in the *Ardeinæ*, is sufficient to satisfy one that it is a very different affair

Fig. 10.—Right lateral view of coracoid and scapula. *Ardea herodias*. Life size from nature.

from the corresponding part of the pectoral arch in such forms as *Sula*, *Phalacrocorax*, or *Pelecanus*. In these latter types the united clavicles arch backwards to meet the carinal angle of the sternum, here to articulate with it, or even as in *Tachypetes* and very old Cormorants to actually anchylose with it. The lower part of the furcula in Herons, is, on the other hand, turned forwards from the sternum, assuming a curve not often seen among birds.

Anatomists have termed the clavicular head in birds, the epicleidium, and this end of the bone, according to Professor Parker, ossifies as a separate piece in some forms, notably the

Passerine birds, and may be compared with the pro-coracoid of reptiles. Not having a young, or rather a sufficiently young enough heron, at hand, I am unable to investigate the pectoral arch with the view of ascertaining how the development proceeds in the case of the forms under consideration.

Professor Owen in calling attention to the relation between the hypocleidium of the clavicles and the carinal angle of the sternum in other birds, says: "The process itself reaches the sternum, and is anchylosed therewith in the Pelicans, Cormorants, Grebes, Petrels, Frigate-bird, and Tropic-bird, also in the Gigantic Crane, and Storks in general." I am compelled to take this statement with a little caution—as it does not always anchylose in the Cormorants, fails to do so in a number of the *Podicipedidæ*, as in Clark's Grebe; and, so far as I am aware, rarely in the *Procellariidæ*; I have one or two exceptions before me; the least tendency to form such a union being seen in the Grey Fork-Tailed Petrel, (*Oceanodroma furcata*).

In all of these forms, however, the hypocleidium is in more or less intimate relation with the anterior border of the keel of the sternum. I have examples where the closeness of the contact is very intimate and requires special investigation to determine whether true anchylosis really exists or not.

Fig 11.

Fig 12.

Fig. 11—Left lateral view of furcula of *Ardea herodias*.

Fig. 12.—The same bone from in front. Both life size from nature, and the same specimen.

This is so, even in *Oceanodroma* and *Colymbus* sometimes. I have several skeletons of the former before me, but have figured one where it was the least so. No doubt these facts accompanied by a lack of good material led Professor Owen to make the above statement. Unfortunately at the present writing, no skeletons of the *Ciconiidæ* are available to me; although even in these birds, if actual anchylosis does not take place, we can at least be well assured from the paragraph I have quoted above, that the clavicles do curve backwards to come in close relation with the sternum, a very different condition being present in the *Ardeinæ*, where their lower thirds curve gently forwards in the manner described above.

*Of the Pelvis and Coccygeal Vertebræ*:—A year or so ago I made a number of anatomical drawings for Professor Coues, these now illustrate his admirable "Key to North American Birds," 2d Edition. Among these drawings I figured the under view of the pelvis of *A. herodias*, the bone now to be described. It is figure 60, in the work alluded to, and as the present paper contains two other views of this pelvis (Figs. 13 and 14), I have intentionally drawn them from the same specimen, which I was so fortunate as to still have by me.

The twenty-fourth vertebra of the spinal column of this heron is the anterior one of the series that becomes incorporated by complete anchylosis with those other neighboring bones which go to form the pelvis Indeed, so far as I have been able to examine, it is this vertebra throughout the *Ardeinæ* that holds this place; it is marked *dl* in my figure in Dr. Coues' "Key."

This *twenty fourth vertebra* possesses a pair of free ribs which have already been described above; its neural spine is continuous with the common median crest of the others behind; and its broad diapophyses meet the under side of the ilia, on either side, to anchylose with them. As in the remainder of the pelvic series of vertebræ, this bone is highly pneumatic, the foramina entering the bones much in the same manner as we found them doing in the dorsal region.

The next four vertebræ behind the twenty-fourth, or the twenty-fifth, sixth, seventh and eighth, throw up apophysial abutments against the iliac walls, to completely fuse with them.

After we pass the twenty-eighth we suddenly meet the pelvic basin proper which is here deep and ample; the apophyses of the three next succeeding vertebræ, or the twenty-ninth, thirtieth, and thirty-first are thrown so directly upwards against the pelvic bones, that they cannot be seen on direct ventral aspect. This is the region of the greatest enlargement of the neural canal, and also the bones through which it passes are here more massive in order to contain that part of the cord from which the sacral plexus emanates. The foramina from which they issue, on either side, are double, being placed one above another. This obtains also in at least four of the vertebræ beyond these and one other behind, making eight in all whose sides are pierced by these double foramina.

Apophysial abutments are again thrown out to anchylose with the pelvic bones above them, by the thirty-second to the thirty-seventh vertebræ inclusive. The longest pair of these came from the thirty-second vertebra, and thereafter grow gradually shorter as we go backwards.

The 'brim of the pelvic basin' is continuous with the processes of the thirty-sixth vertebra posteriorly, while anteriorly, it merges with the posterior border of the transverse processes of the twenty-eighth. This boundary has a rounded and well-defined border in the Great Blue Heron, and is more or less determinable in the majority of birds. When viewed from above, this bone presents a strikingly smooth and unbroken superficies—it is scarcely marked by either crests or ridges, and in my specimen only two pair of inter-apophysial foramina are seen, these being between the last two vertebræ.

Fig. 13.—Superior view of the pelvis of *Ardea herodias*. Life size from the same specimen as in former figures.

Anteriorly, in the median line, the neural spine of the twenty-fourth vertebra is observed to project as a tuberous and notched process.

For some little distance back of this the ilia meet on either side of this common neural crest, sealing over the ilio neural grooves and making one rounded summit for this part of the bone.

The anterior margins of the ilia are notched and scalloped, and bordered by a somewhat deep and slightly raised emargination. Where these bones are broadest in front, the lateral edges are quite sharp, but as the pelvis contracts in width as we near the acetabulæ they become rounded and smooth. The iliac surface, on either side, thus bounded, is at first directed upwards and outwards, but as we approach either actabulum, this surface gradually comes to look almost directly outwards. Ilio-neural grooves exist between the anterior forks of the gluteal ridges, for some little distance, before these latter, and well defined crests, are lost anteriorly (Fig. 13).

Few traces or markings are left upon the inner margins of the post-acetabular surfaces, to define the boundaries which originally existed between the vertebræ and the iliac bones; they are best seen behind. For the most part though, the pelvic roof has become in the adult, one unbroken surface—a very smooth and firmly ossified tract.

The outer angles of the gluteal ridges are rounded and project immediately over the antitrochanter, on either side, from which point each ridge runs almost directly backwards to the hinder margin of the bone. This latter, as a whole, is concave towards the posterior aspect, and from its outer angles the curved and inturned pubic bones may be seen pointing towards each other, their tips some 2 centimetres apart.

Only a limited part of the surface of either ischium can be discerned from this superior view, as these bones behind are nearly at right angles with the overhanging ilia.

Among all the *Ardeinæ* that I have had the opportunity to examine, the post-acetabular surface is about equal in extent with the pre-acetabuler area. In the former the general surface is convex, while in the latter it is concave; the boundary between them I place, in common with Owen, at the line of the gluteal ridge. The post-acetabular surface slopes downwards from a line joining the outer gluteal angles; the amount of which declination can best be appreciated by a glance at my figure of the side view of this pelvic bone (Fig. 14).

Upon lateral aspect the centra of the leading vertebræ may be seen below the eaves of the iliac roof, and some idea gained of the massiveness of the osseous column upon which the pelvis of this Heron is built.

The acetabulum is large and circular, with its floor more than usually deficient, the inner ring nearly equalling in size the outer, while the antitrochanterian articular surface is carried by them both as it passes inwards. Externally this facet looks downwards and only slightly outwards.

The *ischiadic foramen* is large and sub-elliptical; its major axis being parallel to the line of the outer border of the post-acetabular surface, which here arches over it. Posterior to this foramen, the broad part of the ischium is roughly quadrilateral in outline, and for the most part smooth and slightly concave. It is nearly at right angles with the iliac surface above it. In this

Fig. 14.—Right lateral view of the pelvis of *A. herodias*, same bone shown in Fig. 13. Life size.

heron the *obturator foramen* is far from complete or deserving the name of a foramen. Nearly its entire posterior arc is deficient, and the opening thus created, leads into the obturator space, which latter is found beneath the entire lower margin of the ischium, being broadest in front and gradually tapering off behind (Fig. 14).

*Ardea herodias* has a blade-like pubis, of nearly an equal width throughout, though rather wider behind, after it passes the ischium and curves mesial towards its fellow. Just before it does this it is slightly over-lapped by the lower and posterior angle of that bone, or else meets it in a single point of tangency, or, as in the figure, does not quite come in contact with it. Quite a large pneumatic foramen is found beneath the projection of each ilium immediately behind the anti-trochanter.

The vertebral column may be seen in part through the apertures afforded by the acetabulum and ischiadic foramen upon this lateral view. Except at its sacral dilatation, the neural canal as it passes through the vertebræ of the pelvis is small ; it will be remembered that we found it quite so in the dorsal region also.

My specimen of the pelvis, taken from the skeleton of *Ardea candidissima* (a bird of the year), although thoroughly herodine in all of its salient points, it still differs in some of its minor details, from the same bone in *Ardea herodias*. A careful count shows that an equal number of vertebræ are anchylosed together to form the central mass for the support of the pelvic arch,—fourteen in each case, *i. e.*, the twenty-fourth to the thirty-seventh inclusive. This obtains also in the Yellow-Crowned Night Heron, and in both these birds the rim of the pelvic basin departs from and arrives at identically the same segments as described for *Ardea*.

In *A. candidissima*, the ilia do not overreach the twenty-fourth vertebra, although otherwise these bones are comparatively longer and narrower than in *A. herodias*. A greater number of inter-apophysial foramina pierce in double rows the middle area in this heron ; these, however, may be obliterated in older birds.

*Nycticorax* also possesses a true heron's pelvis, and so far as this bone is concerned the differences between it and the pelvis of *Ardea herodias* are of so trivial a nature as scarcely to be noticed on first sight. The principal ones are these : in *Nycticorax* the gluteal ridges and outer angles are not nearly so prominent ; a greater number of inter-apophysial foramina exist upon the dorsal aspect ; the last vertebra, the thirty-eighth of the spinal column, anchyloses with the sacrum, although it projects entirely beyond the pelvis, this one corresponding to the first of the free coccygeal series in *A. herodias* ; the hinder ends of the ischia are cut squarely across and do not apparently project beyond the ilia ; and finally, the obturator foramen is more nearly entire.

I find seven freely articulated *coccygeal vertebræ* in *Ardea herodias* and a pygostyle. *A. candidissima* shows but six, and the pygostyle, but it may be possible that one of these vertebræ has by some accident been lost in my specimen. We saw above in *Nycticorax*, how, in that heron the first one of the series anchylosed with the pelvis, both by its centrum and by the antero-external angles of its diapophyses.

These seven vertebræ in the Great Blue Heron are non-pneumatic, and all but the last three entirely devoid of hypopophyses, and it may be absent on the first of these.

The first five have broad flaring diapophyses, which are entirely aborted in the last segment, and only barely apparent in the one that precedes it.

In calibre, the neural canal is larger than we would be led to expect from the size of that tube as it appears in the last uro-sacral vertebra of the pelvis.

The neural spines are bifid and sub-compressed, while the form of the anterior and posterior articular surfaces of the centra are transverse and flattened ellipses.

Herons being birds with short, weak tails, composed of but a few feathers, we naturally find a correspondingly feebly developed pygostyle.

In *Ardea* this bone has projecting forwards from its lower anterior angle a process nearly as long as half the bone itself. It represents the hypapophysis of the leading vertebra that was absorbed, to form, with probably several others, this compound bone. Very faint traces of another such a process may be seen marking its side further back, and above it, the barest hint of the centrum of the corresponding vertebra. For the rest, the pygostyle is an irregular, quadrilateral plate, less than a centimetre deep, and a little more than one long, measured on its longest diameter; with a round, thickened posterior margin, and upper and lower edges sharpened. A pit marks the flat anterior surface, which continues for a short distance into the substance of the bone, the neural canal of the caudal vertebræ. Other herons have the pygostyle rather differently fashioned from this, though in each instance the leading features are present.

*Of the Appendicular Skeleton. The Pectoral Limb:—Ardea herodias* has a highly pneumatic *humerus*, which in the well prepared skeleton is a snowy-white, and for its size a wonderfully light bone. Nor is the pneumatic aperture of any great dimensions, it being a small sub-elliptical opening at the usual site for this orifice in birds. It differs somewhat, however, in lying in the same plane with the general humeral surface, below the ulnar crest, and not being situate at the base of a pneumatic fossa, in which several openings are usually seen leading to the hollow shaft of the bone. From radial to ulnar side the proximal dilation of the humerus is not nearly as great as we find it in many others of the class. At its summit there is an oval, convex facet for the glenoid cavity.

This is separated from the ulnar crest by a deep intervening valley, which appears all the deeper from the great prominence attained by the crest in question.

The radial crest is, on the other hand quite low, and not unusually developed. It extends down the shaft only to the point where the latter commences to assume the cylindical form. On the palmar aspect of the proximal end of the humerus we have a well defined trench extending across the bone, just behind the ulnar crest and glenoid head. Another fainter one, though pretty well marked in the direction of the shaft, marks out the boundaries of a convex, sub-oval and flattened space, on the lowermost side of the palmar aspect of the proximal end of the bone, which is present in some form or another in this place on the humerus, in a number of the class.

The shaft for the greater share of its length is cylindrical and smooth ; the sigmoid curves it presents in the majority of birds are here well marked. The distal extremity is dilated in the same plane nearly with the proximal end, to give space for the guidance of muscular tendons on the anconal side, which there pass over grooves marking the bone, as well as affording the necessary breadth to support the ulnar and radial tubercles on the palmar side. Above the latter is seen a long, subelliptical depression, running obliquely up from this dilated portion to a point where the shaft begins to assume the cylindrical form.

Albatrosses and some other sea birds, as the Gulls, Auks, and Petrels, the humerus presents a notable "ecto-condyloid" process on the radial side, near its distal extremity (Owen). No trace of such a process as this is found among the herons. So far as I have examined them, I found it, however, on the humerus in the locality just referred to, of *Numenius longirostris*, of *Hæmatopus*, and of other limicoline birds, and presented a figure of the bone showing it in the first mentioned form, in my osteology of the Long-billed Curlew which appeared in the nineteenth volume of the *Journal of Anatomy and Physiology* (Oct., 1884).

The *radius* is a non-pneumatic bone, and like all bones of this character, in the ordinarily prepared skeleton becomes yellow, dark and greasy, owing to the oily constituents of the contents of the shaft gradually oozing through its walls.

This bone, in common with its companion in the anti-brachium, is considerably longer than the humerus. From proximal to distal extremity its shaft is much bowed in the palmar direction.

The proximal end is comparatively little enlarged ; it presents the usual sub-ellilptical facet for the humeral tubercle of the bone of the brachium, on its end, and shaft-wise, the ulnar facet is presented for our examination.

For its length and the general size of the bird, the shaft of the radius is quite slender. In form it is sub-trihedral with the salient angles rounded off.

Its distal extremity, moderately dilated, and compressed from above, downwards, shows on its superior aspect the grooves for the lodgment of the tendons of the hand. A long narrow facet occupies the extreme end of the bone for the *radiale* of the carpus ; this end of the radius curling over in a downward direction, so when articulated in the normal position of rest it overlaps the ulna.

Fig. 28.—Right lateral view of the skull of *Nycticorax violaceus*, 'bird of the year' (July). Life size from nature, from a Louisiana specimen collected by the author.

When these bones of the anti-brachium of *A. herodias* are articulated as in life, I find that the interosseous space, occupies but a little more than the proximal half of the distance between their extremities, while for the remainder, they almost come in contact with each other, being but slightly separated again just before arriving at their distal ends

Usually the *ulna* is quite straight, or has only a slight degree of curvature, but in the present subject it is bowed nearly as much as the radius and very much in the same way. It is hardly necessary to say that in common with the radius and the skeleton of the pinion, that it is likewise found to be a perfectly non-pneumatic bone. Its shaft is about two-and-a-half times the size of the radius, but instead of being sub-trihedral in form, it is nearly cylindrical.

Two rows of quill-knobs are distinctly seen upon its length, one on the ulnar and one on the palmar aspect; the former being the more strongly marked.

The shaft decreases in size gradually from the proximal to the distal end, very imperceptibly from the middle of the bone, on. A nutrient foramen is seen on the anconal aspect at the proximal part of the middle third.

The carpal end shows the usual trochlear surface, and the facets for the *radiale* and *ulnare* of the wrist. Proximally, the enlargement is much greater, in order to afford sufficient breadth, to make room for the extensive excavations that are found at this end, to articulate with the radius and bone of the brachium. The olecranon is but feebly developed and tuberous. Measurements taken from these bones in an adult specimen of *Ardea herodias*, shows the humerus to be 19 cms. long; the radius 22. and the ulna 23.1, which goes to show that the brachium and anti-brachium are proportionately balanced as to their respective lengths. Both of the carpal elements are present, the *radiale* and the *ulnare*. They are of good size, articulate as in most birds, and are fashioned after the most usual pattern assumed by these bonelets.

The *carpo-metacarpus* makes up in length in this heron what it otherwise lacks in breadth. It measures 10.3 c. m. long, while across the widest part above it is but 1.8 c. m.; this latter measurement being from superior tip of pollex metacarpal directly across the bone to outer edge of trochlear surface.

The first metacarpal, anchylosed as usual at the upper and anterior aspect of the bone, is very short, slightly bent anconad, and directed rather upwards as a tuberous process. Beneath, it supports the extensive convex articular facet for pollex digit, which latter is long and somewhat laterally compressed. It bears a diminutive facet at its distal extremity, and appears as though it might have had in life a claw there, which has been lost in my specimen. Nitzsch, who examined many groups of birds to investigate among others this point, places the Herodiones in the category of birds in which he discovered it to be present. So on the authority of this eminent anatomist I believe we may safely say that our subject will be found to possess such a claw.

For its entire length the main shaft of this bone is very straight, and such part of it as is free from contact with other bones above and below, is subtrihedral in form and devoid of particular character.

Showing a considerable transverse dilatation at its proximal extremity, the third metacarpal soon quits the shaft of the second, to become much smaller and rounder, to be found parallel to it, until within a short distance from its lower end, where they are again connected by bone.

At the proximal extremity of this carpo-metacarpus, we find a broad trochlear surface, contributed in the usual manner by the *os magnum*, one of the carpal bones free in the wrists of subadult birds. As in the majority of cases all the sutural traces of this union, have with the growth of this heron become obliterated.

Fig. 29.—*Nycticorax violaceus* juv. Superior view of skull. Same specimen as shown in Figure 28. Life size.

Upon the palmar aspect, just below the superior convex margin of this trochlear surface, at the head of the index metacarpal, we observe projecting forwards a small stumpy process.

The distal end of the carpo-metacarpus in the adult *Ardea herodias* is almost entirely occupied by the two articular facets for index and middle digits. A notch divides them. In the case of the first, the proximal phalanx is a long bone (3.8 c. m.), with a posterior blade-like expansion. This latter is not very broad, being thick, and unpierced by foramina, as we sometimes see it in the Gulls and other water birds. A long, pointed subtrihedral joint succeeds this one, which in turn seems to have a facet upon its distal extremity, either for a claw or another minute joint, such as we find among the Ducks and Geese, but in my specimen it is missing. The third metacarpal supports a digit composed of a single subcompressed, narrow phalanx, nearly two centimetres long.

Taken in connection with what Nitzsch has given us upon the subject, I believe the formula for the manus of the Herodiones will be found to be—pollex metacarpel, with a digit composed of two phalanges ; index metacarpus, with a digit of three phalanges ; and middle metacarpus with a single phalanx to its digit.

So far as the material goes that I have been able to examine, the pectoral extremity among the *Ardeinæ* offers no very striking differences. As a good illustration of the slight departure that is made from a common plan among these Herons, no better example could be offered than the series of bones shown in figures 35, 36 and 37, being the right humerus from *Ardea herodias*, *Nycticorax* and *A. candidissima*.

*Of the Pelvic Extremity.*—After the most careful examination of the material at hand, I find it is only in the femur of *Nycticorax* that pneumatic foramina exist. These are exceedingly minute, though they may be detected without the aid of a lens just over the border of the anti-trochanterian facet on the posterior aspect of the bone. In *A. herodias* and *A. candidissima* the *femur*, as well as all the other bones, composing the skeleton of this limb, are absolutely non-pneumatic.

Fig. 30.—*Nyctiaorax violaceus.* juv. Its mandible from above: life size ; same specimen as figures 28 and 29.

Our Great Blue Heron has a femur fully as long as its pelvis omitting the free, posterior end of the pubis. Its head and neck make nearly a right angle with the shaft, the former being hemi-globular and much excavated for the ligamentum teres, while the latter is short and thick. At the summit of the bone the anti-trochanterian facet is broad and extensive. From before, backwards, its surface is convex ; in the other direction, that is from the head to the trochanter, it is concave, becoming gradually wider as it approaches the latter.

The trochanterian ridge does not rise above this articular surface to any perceptible degree, but becomes rather prominent as it passes down the shaft for a short distance on its outer and anterior aspect.

On the outer and proximal end of the femur, the trochanter major is broad and nodular. The shaft below this point, to where it begins to expand for the condyles, is nearly straight, and quite cylindrical. Its muscular lines are distinct and raised; on the posterior aspect, above the middle, the nutrient foramen is to be seen. It opens in a direction obliquely from above downwards.

Just above the anterior ridge of the external condyle, I find in all herons, the antero-external aspect, a prominent and elongated tubercle. It has to do with muscular attachment, and one of the muscular lines is deflected from its course to run into its upper end.

The condyles of this bone are strong and massive. The articular surface of the inner one is broad behind, and so far produced in this locality as to render the popliteal depression appear more than usually concave and excavated. Above each condyle behind is seen a well marked tubercle, with pits on their outer sides for the insertion of lateral ligaments and muscles. The external condyle has the usual fibular groove, deeply cleft and carried down well nigh its base, behind; it is more prominent than its fellow, though not as broad. Between them, the inter-condyloid fossa is moderately deep, rather wide, and carried up on the anterior aspect of the shaft as a "rotular channel" of like dimensions, though not mounting as high as it does in some birds. Of these two condyles, the external one is rather the lower, the femur being held in the vertical position.

I fail to find a *patella* present in any of the *Ardeinæ;* in *Nycticorax* a thickening in the ligament takes place at the usual site of this sesamoid in other long-legged birds where it is found, but this ligamentous enlargement is entirely devoid of any osseous deposit.

The *tibia* of *Ardea herodias* as we might know is a very long bone, and in every particular typical as found in Herons generally. Viewed directly from above, on its proximal end (Fig. 21), we observe that it has a roughly quadrilateral outline, its general surface sloping towards the fibular side.

The intercondyloid tubercle is prominent, and situated rather external to the centre of this surface, while anteriorly it is bounded by a low cuemial crest.

Regarding the shaft from in front (Fig. 23), we notice that the pro- and ectocnemial ridges are but moderately developed, and very soon subside into the shaft below. A wide valley is between them, and the inner one or procnemial ridge is vertical to the shaft and exactly divides the inner surface of it from the anterior.

All about the head of the tibia the articular summit projects over with its broadly rounded margins.

*Fig. 31.*

Fig. 31—Left lateral view of sternum of *Ardea candidissima.* Life size from nature.

The "fibular ridge" extends down the tibial shaft on its outer side but a comparatively short distance. It begins above at a point opposite where the ectocnemial ridge merges into the shaft. Behind, a longitudinal concavity fairly defines its extent from the posterior surface of the tibia; in front, the anterior surface of this fibular ridge lies in the same plane with the anterior surface of the tibial shaft.

From proximal to distal end this shaft is as straight as any long bone that I am familiar with; it is only just before we arrive at the condyles below that we notice the slightest disposition in the world to bend backwards.

*Fig. 32.*

Fig. 32.—Left lateral view of sternum of *Nycticorax violaceus.* juv. life size; from the same specimen as former figures used for this bird.

For its entire length behind, the surface is cylindrical; this is entered into by both the lateral aspects, while anteriorly it is flat, and only round at all for a limited part of the shaft about at the junction of middle and upper thirds. This flat anterior surface above, looks directly forwards, and this is the case also above the tendinal bridge, but as we ascend the shaft from this latter point, it gradually turns towards the outer aspect, where finally it is limited by a raised line that descends on this side from the fibular ridge, and merges at last into that part of the shaft which is subcylindrical, at juncture of upper and middle thirds.

At the distal extremity, the shaft enlarges but very slightly, and just sufficient to afford a base for the condyles, which here project, in consequence, well out in front of it, both before and behind, more particularly in the former direction (Fig. 24).

The "tendinal bridge" though present, is not nearly so well developed as in some other birds, and in my specimen of *Nycticorax* a "bird of the year" it is not united in the middle, it being simply represented by a triangular process on either side, with their bases in the margins of the excavation, and their apices opposite and nearly touching each other. A tubercle occurs above the outer condyle where this bridge abuts on that side, which is its lower one, it spanning the tendinal groove rather obliquely.

The inter-condyloid depression is wide, deepest in front, to become narrower and shallower behind, where it ceases as the shaft commences.

Viewed anteriorly, the outer condyle is the broader, extends higher on the shaft, but projects no further in front than the inner one. This latter, slightly encroaching on the inter-condyloid space, is excavated by a well defined subelliptical pit, which is better marked in the Night Herons, though present in the *Ardeinæ* generally.

Viewed from behind, these condyles of the tibia in *Ardea* mount to points about opposite each other on the shaft. Here, however, the inner condyle is the broader, and rather more prominent above.

Upon lateral aspect these condyles are reniform in outline with the convex surfaces below; and from above, downwards, the outer is the deeper of the two.

In my Osteology of the North American Tetraonidæ I described the method of ossification of the cnemial crest of the tibia in the young of *Centrocercus urophasianus*. In the memoirs in question I gave a figure showing this development, which in brief consisted in a large osseous segment engrafted upon the bone, at the future site of the cnemial crest and upper halves of the pro- and ecto-cnemial ridges, all of which it formed, but left no trace of such a develepment in the adult fowl.

A re-examination of this state of affairs convinces me, that in the bird alluded to, the description is correct in every particular, and my only regret is that I have not at this moment the proper material to investigate whether or no a like method of development goes on in the young of the Herons.

As for the distal extremity of this bone, it also has received no little attention generally, but in particular the young of our present subject has been ably investigated at the hands of Professor Morse.

It was through his studies of the tibia and tarsus of immature individuals of various species of *Ardea* that this distinguished zöologist was principally enabled to demonstrate the presence of the intermedium in the class birds. Professor Morse's researches have proven, I think, beyond doubt, that the "ascending process of the astragalus" of Huxley agrees with the "pretibial" of Wyman. Further, this segment ossifies from a separate centre of ossification, and as such constitutes in the avian tarsus a third bone of the proximal row, which corresponds with the *intermedium* of the Reptilia as described by Gegenbaur. No marked suspicion exists of the presence of any such bone in the adult, in any of the *Ardeinæ*, it having been completely absorbed by the tibia, and every vestige of its original limits obliterated.

The *fibula* of the Great Blue Heron is a very much aborted bone, both in comparison with many other birds and with the size of its own tibia (Figs. 19 and 20).

The upper surface of its distal end is devoted entirely to the facet for articulation with the condyle of the femur. Below this the bone is compressed from side to side, and produced from before, backwards. Then rapidly contracting it presents a roughened surface intended for ligamentous attachment to the fibular ridge of the tibia. Near this we see the tubercle for the insertion of the tendon of the biceps. The remaining length of the fibula becomes almost needle-like in its dimensions, and makes no osseous connection with the tibia whatever, passing but little below the upper third of its shaft, which when the bone is removed shows no evidence of its contact, more than the roughness of the fibular ridge.

*Ardea candidissima* has a fibula that agrees in all respects with the one I have described for the Great Blue Heron. In *Nycticorax* it differs in one important particular, and this is, that after passing its articulation with the fibular ridge of the tibia, its almost thread-like dimensions are carried well below the middle of the shaft of the leg-bone to unite with it by ossification, for at least a third of this part of its length.

Next in order we have to notice the *tarso-metatarsus*. The differences that this segment of the lower extremity exhibits among the various herons, seem to be scarcely worth the mention. So I expect a description of the bone as it is found in *Ardea herodias*, will answer with sufficient exactness for the group.

Different views of the tarso-metatarsus are shown in figures 15, 16, 17 and 18 all drawn from an adult specimen of the Great Blue Heron.

A very prominent tubercle occupies the anterior part of the superior surface of the proximal extremity. It stands between

Fig. 33.

Fig. 34.

Fig. 33.—Left three-quartering view of furcula of *Nycticorax violaceus*, juv.

Fig. 34.—Same view of the *os furcula* of *Ardea candidissima*. Both life size from nature.

the two elliptical concavities intended, when articulated, for the condyles of the leg-bone. The margins surrounding the extremity are raised at the sides and sharpened. Posteriorly, we can also see from this view, the three processes composing the *hypotarsus*. Of these the innermost one projects the farthest backwards, as well as extending the greatest distance down the shaft. The outermost one of the three is the smallest, being just about half the size, in height as in length of the innermost one. The middle one, falls between these two so far as its height is concerned, but it is as long as the innermost one (Figs. 17 and 18).

In order to support this great, tendon-grooved hypotarsus, and broad articular surface, the shaft of the bone at this end is proportionately enlarged. It grows gradually smaller, however, as we descend, being of the least calibre in the lower third, when it again enlarges transversely to support the trochleæ for the digits. The upper half of the bone is flat both posteriorly and at the lateral aspects. In front it is longitudinally excavated down the middle, beginning where it is the deepest, just below the inter-condyloid tubercle. These surfaces are exchanged for the sub-elliptical shaft as we gradually pass to its lower half, the major axis being transverse.

At the base of the excavation above, a few millimetres below the anterior crest of the summit, we find the shaft pierced by the foramina, placed side by side. The innermost and larger one of these passes rather obliquely through the the bone to make its appearance, rather larger in size, just inside of the hypotarsus.

Considerably smaller, its companion pierces the tarso-martarsial shaft, still more obliquely downwards, to make its exit as a foramen of diminished calibre on the opposite side of the hypotarsus. The posterior opening of this latter one is seen in Figure 15.

Viewed from in front, the trochleæ present the following points for examination : the mid one extends the highest on the shaft, and projects beyond the others anteriorly. It is distinctly grooved down its middle, and descends the lowest. The inner one is the broadest and is perfectly smooth in front, being but slighly grooved behind, while the other two are decidely so. Finally, the outer trochleæ is also smooth in front, and does not descend as low as either of the others. Between this one and the next the usual foramen pierces the bone, low down in the groove between them.

It will be seen that these trochleæ are so placed as to be slightly convex forwards, and in a less degree concave behind, where they come up to nearly the same points on the shaft, the middle one being rather the lowest. Moreover, the mesial grooves that mark them are here carried up to their very terminations. This posterior aspect of the distal extremity also shows the foramen for the anterior tibial artery in full view, above these trochleæ, and on the inner side above it, a circular facet for the first metatarsal.

These three long bones of the pelvic extremity of *Ardea herodias* have the following measurements in the adult : the femur, measured from the highest point on the trochanterian ridge to the lowest point on the outer condyles, is 10.5 c. m. long, the tibia, 24.5 c. m. and the tarso-metatarsus, 17.8 c.m. long. Measuring from the highest point on the intercondyloid tubercle to the lowest point on the mid trochlea. We may add here the length of the fibula which is but 9. c. m, being one and a half centimetres shorter than

Fig. 35.—Anconal aspect, proximal extremity of the right humerus of *Ardea herodias*.

Fig. 36.—Right humerus of *Nycticorax violaceus*, juv., anconal aspect.

Fi5, 37.—Right humerus of *Ardea candidissima*, anconal aspect. All three bones life size from nature, from specimens in the author's collection.

the femur, and fifteen and a half shorter than its companion bone in the leg.

The first metatarsal is a free bone, with a peg-like shaft and enlarged lower extremity. Somewhat of a dilatation takes place at its proximal end which bears a circular facet on the lateral aspect, to articulate in life with the surface described above on the tarso-metatarsus. Thus it is that this bone is so mobile, and can be thrown backwards to a considerable distance. Below, it bears a trochlea for the rear phalanx of hallux, which reaches higher on its shaft on the digital side of the bone, being faintly grooved on the other. The entire length of this segment is 1.7 centimetres.

At the proximal end of the first phalanx of hallux, the trochlear surface is far more extensive than its opposed surface on the first metatarsal, being fully half as broad again. The shaft is rather slender, gently curved throughout, convex upwards, and subcylindrical on section. Its distal trochlear surface is principally on the under end of the bone, rather narrower transversely, and shows a shallow median longitudinal groove. The oval sides of this extremity are marked by pits for ligamentous attachment. It measures in extreme length 4.6 centimetres, being the longest phalanx of the pes.

Its osseous claw is rather more than moderately curved, shows the usual trochlear surface and the tubercle for tendinal insertion. The distance from this latter point to the apex measures 1.6 centimetres. Both the convex surface above and the concave surface beneath is uniformly rounded off, while the bone is laterally compressed. A groove distinctly marks it on either side, but is not quite carried to the apex.

Second digit has three phalanges including the ungual one; the proximal phalanx has all the characters as given for first joint of hallux, it, however, is distinguished by a prominent tubercle to the inner side of the articular surface for the trochlea of tarso-metatarsus. The bone is rather stouter and somewhat shorter. The second joint is a still shorter and a slighter bone; its proximal trochlea is concave from above, downwards, very slightly convex in the opposite direction. The shafts of these bones are not curved to such a degree as we found the shape of first joint of hallux to be, and the proximal ones are always the straightest. Agreeing even in minor details, the ungual phalanx of this second digit is smaller than the one found in the first toe, but shows about the same amount of curvature. These three joints measure from proximal to distal one, respectively 4.4, 3.1 and 1.1 centimetres; the ungual joint being measured as I measured the bony claw of the first digit.

The four joints of the middle or third digit have the general characters as given for these phalanges above. Measuring them in the same way and in the same order, I find that the proximal phalanx to be 4 centimetres long; the next 3.9, the next 2.1; and the ungual one, measured as before, 1.1 centimetres long.

Outer digit has five joints agreeing in the main with the other phalanges of the toes of this heron's foot. They measure, in the order as given above, from proximal to last one, 2.9, 2.8, 1.9, 1.7 and 1 centimetre long. Of course the actual length of these ungual measurements will be found to be rather more than those I have given, but it must be remembered that I only present the length of the chord from the tubercle on the under side of the proximal extremity to the apex of the joint.

Herons possess no special ossifications other than those I have mentioned, that I am aware of, in their skeletons.

They have, in addition to their general structure, three peculiar external characters in common with a no less remotely related group of birds than the *Caprimulgi*. Coues, in characterizing the Night-jars, says: "Besides the semi-palmation of the feet, there is another curious analogy to wading birds; for the young are downy at birth, as in *Præcoces*, instead of naked, as is the rule among *Altrices*." (Key, 2d Ed., p.448.) This author does not mention, in the same connection, the third character, no doubt it having slipped his mind at the moment when the above quoted paragraph was penned. It is, that both the *Caprimulgi* and the *Ardeinæ* possess in common, that very rare character,—the *true* pectination of the inner margin of the claw to the middle toe of pes.

Morphologists seem to be of one opinion as to the position held by the *Ardeinæ*, with relation to other groups of birds, after a consideration of the osteological and other anatomical characters they present.

Parker says in the *Pelagomorphæ* the charadrian type reaches its culmination; yet the most exquisite forms, such as the Egrets and smaller Bitterns, and the most gigantic, as the Adjutant, are evidently specializations of a type similar to the pluvialine *Schizognathæ* (Ency. Brit. 9th Ed. Art. Birds).

The palatal structure of the *Schizognathæ* almost imperceptibly merges into the desmognathous type of skull, while, as in *Crax*

*globicera*, it is likely to make its appearance in the less complicated types of palates in the *Gallinæ*.

Then again, another form that approaches the desmognathous type of palatal structure is *Rhinochetus jubatus*. This bird, the well known Kagu, has received the able attention of Parker, and an exhaustive account of its osteology appears in the sixth volume of the Transactions of the Zöological Society. Its cranial characters bring it quite near the Night Herons, on the confines of the *Gruidæ*, where nearest approached by the *Ardeinæ*. Coues places the Cranes, Rails, and their allies in an order *Alectorides*, and says of them : "The *Alectorides* are schizognathous in palatal structure. The nasal bones are schizorhinal in the Crane type, holorhinal in that of the *Rails*. The angle of the mandible is truncate. The maxillo-palatines are not spongy, but thin and laminate. There are normally no basipterygoid processes. The sternum is typically long and narrow, and may be entire, or deeply notched ; it is sometimes excavated to receive folds of the windpipe. There are two carotids ; and two intestinal cœca are present." "While the general pterylosis is not peculiar, the *Alectorides* normally lack the powder-down tracks so characteristic of Herons and their allies. As to the classificatory muscles of the thigh, all five are present nearly throughout the order ; exceptionally the femoro-caudal or its accessory is wanting. These normally præcocial and ptilopædic (with whatever exceptions) birds are more sharply distinguished from the perfectly altricial *Herodiones* than they are from the completely præcocial and ptilopædic *Limicolæ*, with which latter, in fact, the *Alectorides* are directly connected through the Bustards (*Otididæ*) and the Thick-knees (*Œdicnemidæ*)—the line between the two orders being probably to be drawn between these two families" (Key to N. A. Birds, 2d Ed. p. 665).

In the first part of these osteological studies of the *Ardeinæ*, I stated that it was my intention to close the present part (Part II) with some "supplementary notes," to be incorporated with my review of the characters of the skeleton in *A. herodias* and some representative of the Night Herons. This plan, however, I will change a little, and contrast in a general "synoptical table" all the characters of any importance that I can obtain from the skeletons of the various North American herondine birds before me, especially from those representing the sub-family under consideration.

Five years ago, when this paper was first written, I had but few skeletons of herons in my possession, all belonging to my private cabinet; since then, as I have elsewhere stated (in Part I), I have been permitted to examine a number of herondine skulls in the collection of Mr. Lucas, as well as a few belonging to the U. S. National Museum, all of which material has been of the greatest service in the present connection, and for the use of which my thanks are again tendered.

It was these additional facilities for comparison that induced me to depart from my original intention, and enabled me at the same time to present a more complete synopsis of the comparable characters in the skeletons of our *Ardeinæ*, the same being herewith subjoined.

*Synoptical and Comparative Review of the chief Osteological Characters of certain species of North American Ardeinæ.*

1. In all Herons of this group the superior osseous mandible is of a subpyramidal form, with its base merging into the skull and its apex at the tip of the beak; and with three sides, the angle of the culmen being rounded off, the other two angles cultrate. In length it is a little less than twice as long as the remainder of the skull, being notably *shorter* in some of the Night Herons than it is in the genus *Ardea*.

2. Osseous internasal septum very incomplete or altogether absent.

3. All are acutely holorhinal birds.

4. All have (in the dried skull) a moderate movement at the cranio-facial hinge; best marked in the Night Herons.

5. Ethmoid much swelled; broad and spreading under the frontal region; and truncated transversely in front, just posterior to the line of the cranio-facial hinge.

6. Pars plana very feebly developed both in *Ardea* and the Night Herons. Fails to meet the inferior and backward extending process of the lacrymal of the same side.

7. Very large, spongy maxillo-palatines, lofty and parallel to each other in the rhinal chamber, attached to nasals and premaxillary by bony union. In some specimens they may come in contact with each other mesially, or they may have the anterior part of the vomer resting upon their hinder ends. In *Ardea* they are nearly all of a bony spongy tissue, (cancellous). In *Nycticorax*

they are generally overlaid with compact osseous tissue, and cancellous internally.

8. Vomer is a single plate; deep, sharp and produced in front; doubly carinate above, with the two carinations curled over outwardly so as to create a longitudinal trough upon that aspect; united with palatines behind and free anteriorly. Inferiorly it shows its original bifurcatory form, with greater or less distinctness.

9. Palatines are doubly carinated longitudinally; inner keels being in close contact on the halves towards the rostrum (a contact that may be true anchylosis in very old individuals). Anteriorly they are horizontally flattened and merge with the premaxillary and surrounding bones. The posterior angles of their outer carinations bluntly pointed and not prominently produced. Pterygoidal heads extensively in contact, and above unite to form a groove for the rostrum.

10. A post-maxillary present (?).

11. Basi-pterygoid processes absent (negative character).

12. Quadrates very large; the foot of either having four facets for the mandibular articulation.

13. A lacrymal bone is very large, and articulates with both nasal and frontal of its own side. In *Ardea* its infero-produced portion is roughly parallel to the maxillary below it. In *Nycticorax* it makes a wide angle with the same bone, the anterior end of its infero-produced portion being much elevated.

14. Inter-orbital septum shows one large vacuity which includes the optic and other small nerve foramina near it. In *Ardea* the foramen for first pair of nerves, generally very large. In *Nycticorax n. nævius* these are smaller. In *Nycticorax violaceus* (adult) they are *very small* indeed, and just allow the passage of the nerve.

15. Three jutting processes on lateral aspect of cranium.

16. In *Ardea* and *N. n. nævius* the crotaphyte fossæ are separated by a considerable longitudinal median *line*. In *N. violaceus* it is by a tract of some width.

17. Foramen ovale, lateral.

18. In *Ardea* mandibular angle *obliquely* truncated. In *Nycticorax* mandibular angle *vertically* truncated (least obliquely in *A. virescens*, least vertically in *N. n. nævius*). As a negative character we find the mandible in all *Ardeinæ* without a ramal vacuity.

19. There are 44 vertebræ and a pygostyle in the vertebral column of the *Ardeinæ*. In all, the dorsal series are free; in all, the 24th to the 37th inclusive are anchylosed with the pelvic bones; in all there are 7 caudals. In *Ardea* the 18th and 19th vertebræ bear *free* ribs, and all seven caudal vertebræ are free. In *Nycticorax violaceus* the 17th, 18th and 19th vertebræ bear free ribs, and the anterior caudal vertebræ anchyloses with the sacrum. The *pygostyle* is comparatively small. The epipleural appendages of the ribs are small and free.

20. Sternum of good size; its manubrium prominently developed; broadly 2-notched; four articular facets on either side for costal ribs; carina rather deep, its lower border convex and nearly the arc of a circle; dorsal aspect very concave; coracoidal grooves decussate; costal processes broad; one large pneumatic foramen in the median line above, just over anterior border.

21. Coracoid and scapula non-pneumatic; coracoid very broad below, antero-posteriorly; compressed from side to side above; scapular process small; slight differences in the two bones, at their sternal ends, due to their crossing each other. Scapula broad anteriorly, much compressed from above, downwards; apex rounded; blade rather long, not truncate, but tapers gradually to the end. Furcula non-pneumatic; upper half of each limb convex anteriorly, the reverse below; when articulated with coracoid, nearly reaches the scapula at the inner anterior angle of its head; hypocleidium has a superior and an inferior process.

22. Pelvis is rather massive; pre- and postacetabular surfaces about equal; ischic foramen large; obturator foramen opens largely into obturator space; ilio-neural grooves sealed over anteriorly; one pair of free ribs articulate with the pelvis.

23. The humerus is the only pneumatic bone of the pectoral limb, the periphery of the orifice being in the general surface; remainder of limb well proportioned. Bones of pelvic extremity long and straight-shafted, except the fibula, which is short. In *Ardea* fibula short and free below. In *Nycticorax violaceus* fibula long and anchylosed with tibio-tarsus below.

24. Tendinal osseous bridge at lower end of tibio-tarsus exists and is thrown nearly square across the groove.

25. The hypo-tarsus of the tarso-metatarsus 3-crested, graduated in size, the outer being the smaller; the tendinal grooves pass between them.

26. Pes composed of well-proportioned phalanges, arranged on the plan of 2, 3, 4 and 5 joints to 1–4 toes respectively.

In closing this synopsis of my observations upon the osteology of the *Ardeinæ*, I may add that I have seen enough to convince me that we stand much in need of careful comparisons of the skeletons of more extensive series of specimens of our North American *Herodii*; and especially should the skeleton of *Botaurus lentiginosus* be compared with *B. exilis*, and these with the skeletons of some of the forms I have noticed above. Indeed, a very careful comparison of the entire structure of this very homogeneous group would not be labor altogether in vain or misplaced.

All of the forms of our North American representatives of the genus *Ardea* have the skull very much alike, except, of course, in the point of size. In *Ardea virescens* and *Nycticorax n. nævius* the skulls are notably very much alike, no pronounced characters, in fact, distinguishing them; while on the other hand *Nycticorax violaceus* has a skull that is at once seen to be distinguished from the skull in *Ardea* by its greater average breadth; its comparative much shorter beak; by the form of its lacrymal bone; by the difference in the amount of interspace between the crotaphyte fossæ; and by the minute foramina for the exit of the first pair of cranial nerves as compared with the large vacuities there in *Ardea*, finally by the vertically truncate posterior ends of the mandible, they being obliquely so in the latter genus.

The form of the *lacrymal bone* in these birds is an interesting character, for whatever other morphological differences may exist between the representatives of the genus *Ardea* and *Nycticorax*, we can always distinguish the skull of the former from any of the latter, so far as our North American species go, by it alone. This difference pertains to the lower part of the lacrymal as set forth in my description above (compare figures of skulls of *Ardea* and *Nycticorax* illustrating this memoir).

While engaged upon the present paper, I have had before me skulls of *Cancroma cochlearia* and other foreign heron-forms, for which my thanks are due to the U. S. National Museum, but any allusion to them here would be a passing beyond the limitations of the scope of my present work, and it has been my only aim here to record a few of the comparative osteological characters of our Herons, as offering a chapter that may be both extended and improved upon, some time in the future, and no doubt by abler pens than mine.

## Figures and Tables

**Fig. 9. f1:**
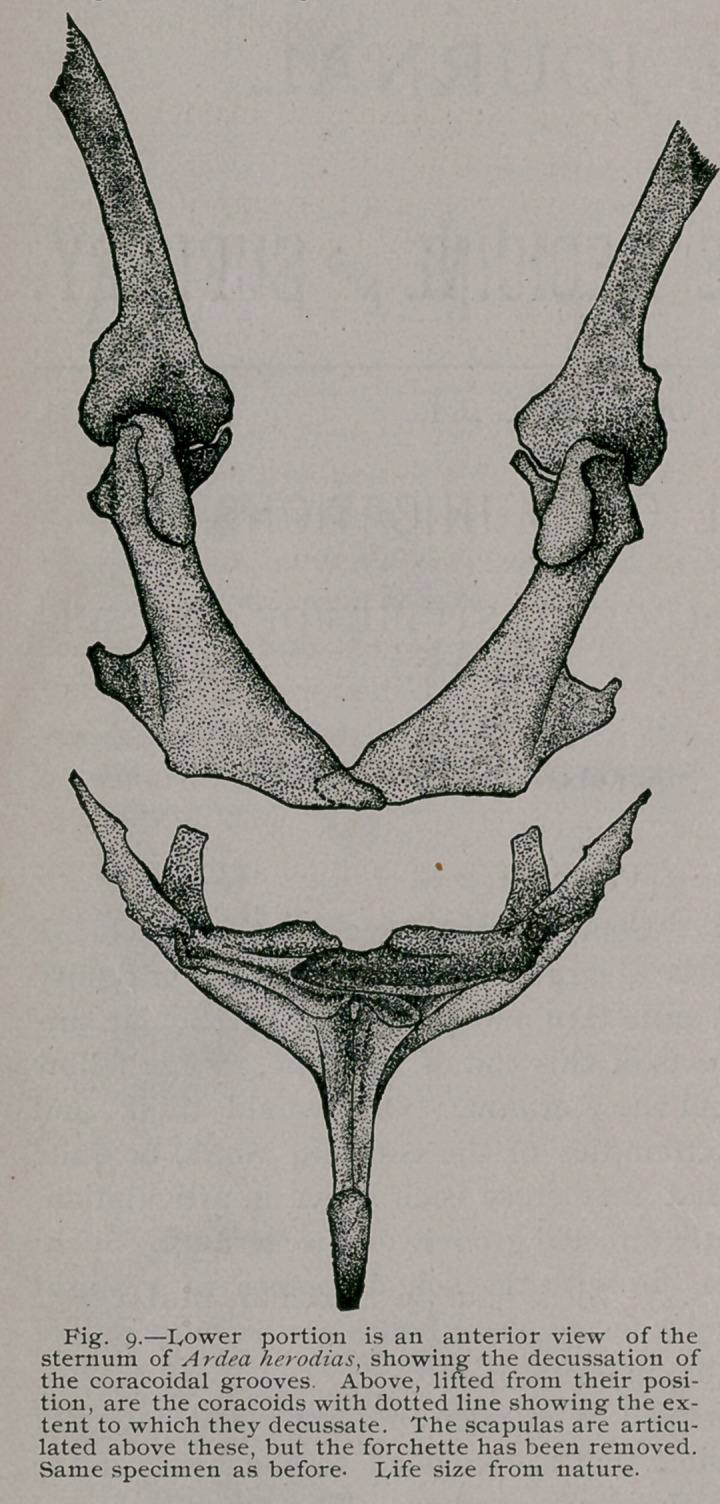


**Fig. 10. f2:**
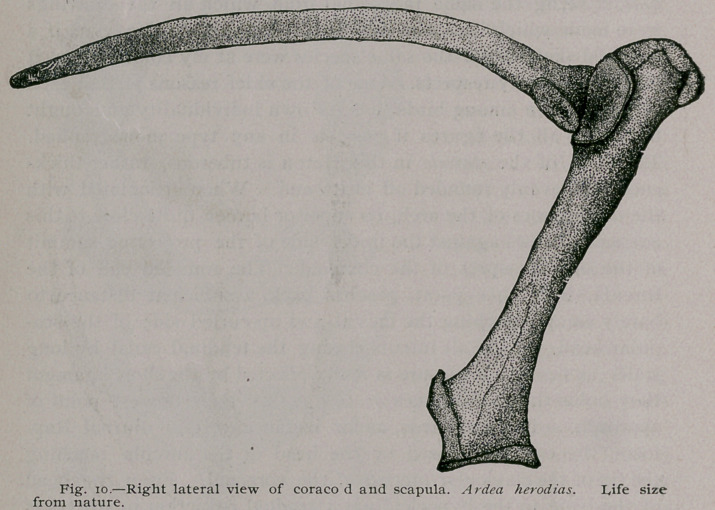


**Fig 11. Fig.12. f3:**
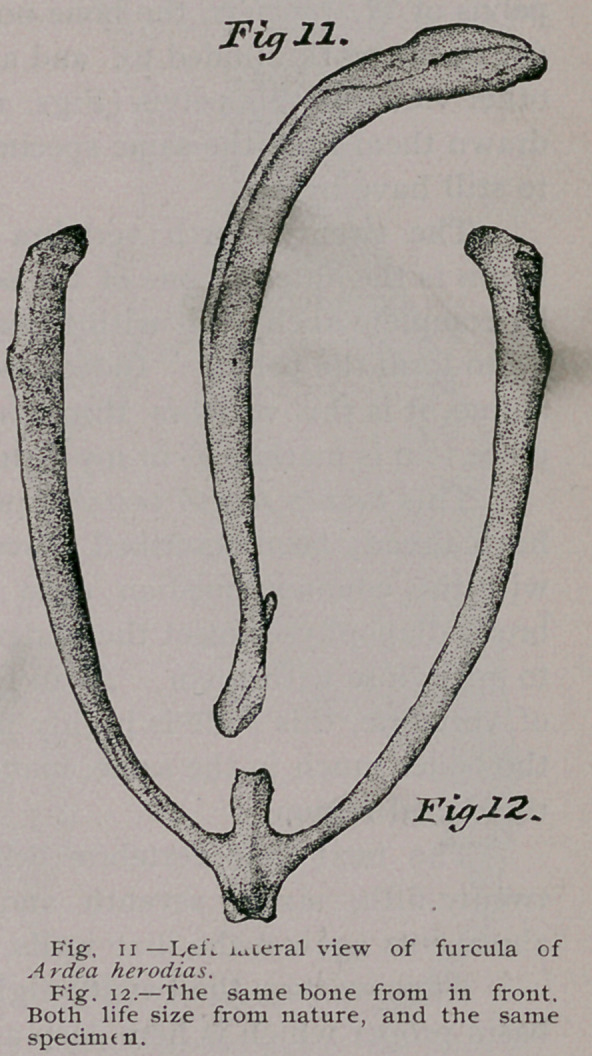


**Fig. 13. f4:**
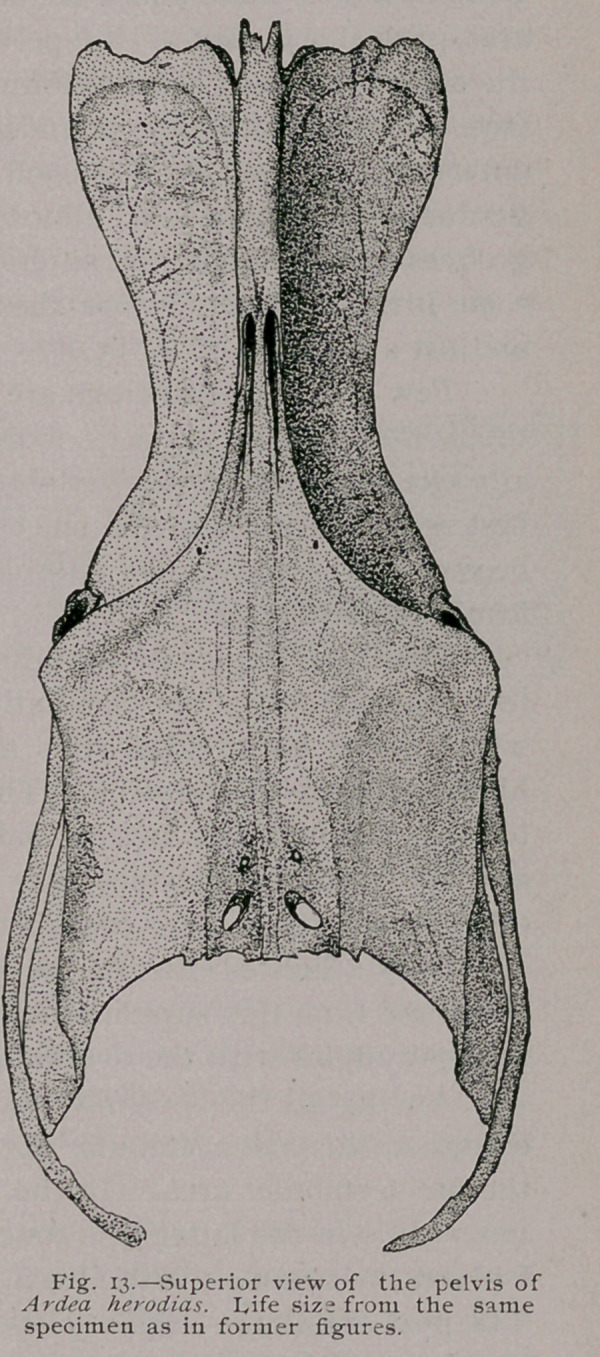


**Fig. 14. f5:**
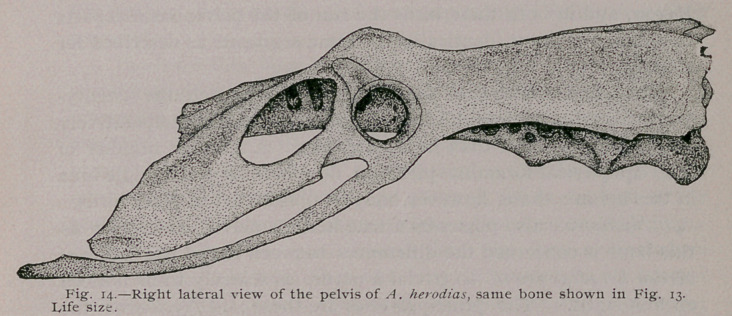


**Fig.15. Fig.16. Fig.17. Fig.18. Fig.19. Fig.20. Fig.21. Fig.22. Fig.23. Fig.24. Fig.25. Fig.26. Fig.27. f6:**
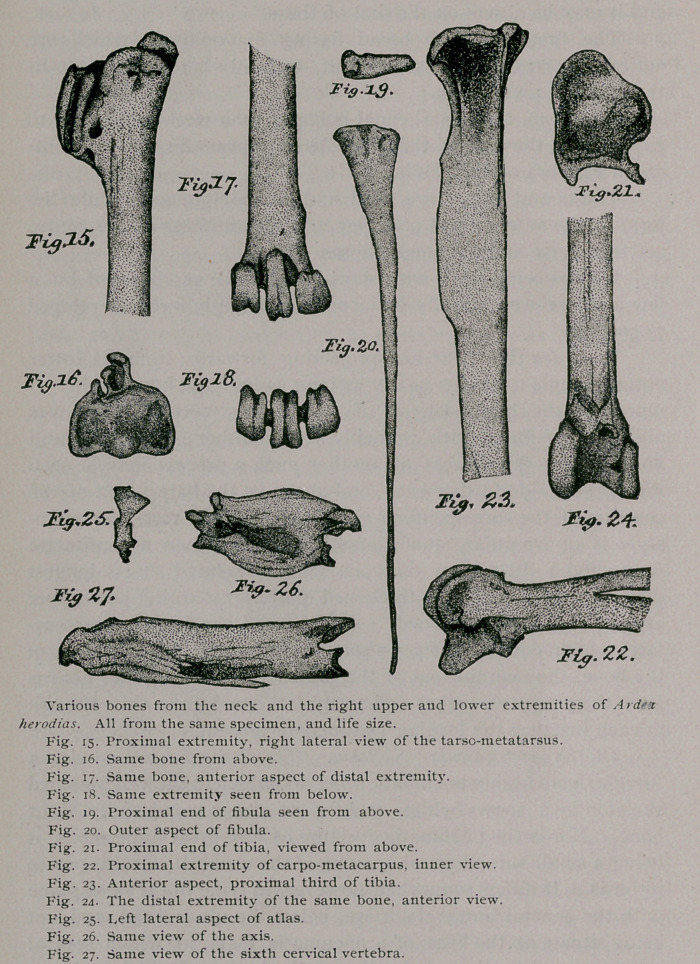


**Fig. 28. f7:**
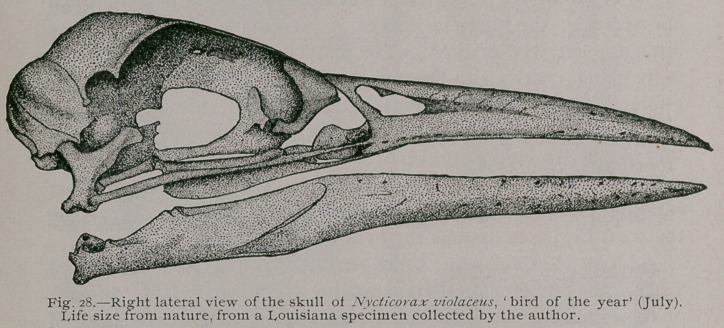


**Fig. 29. f8:**
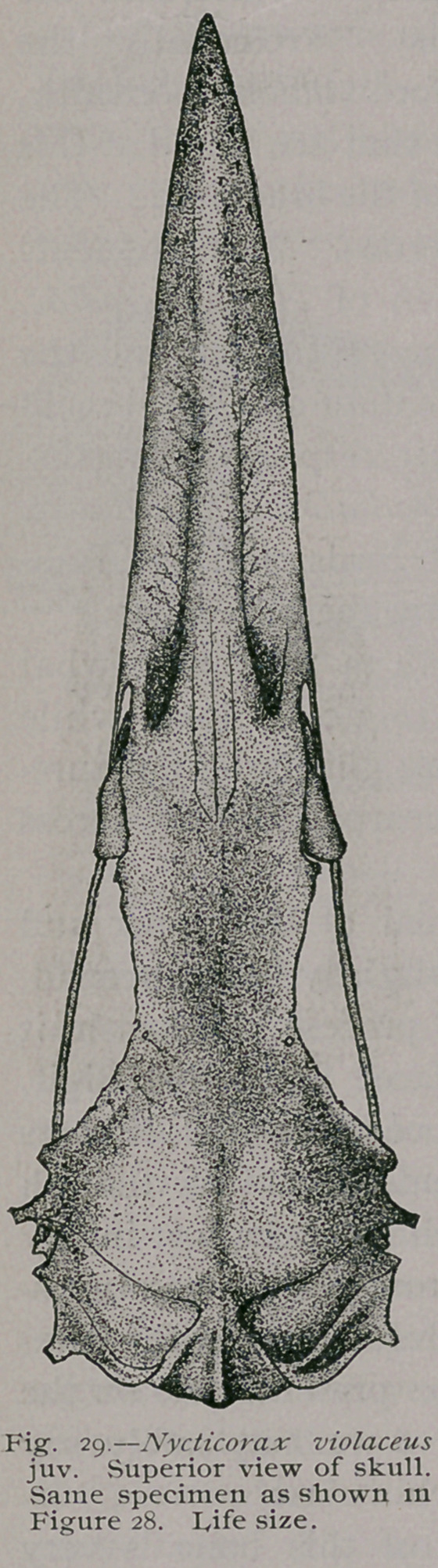


**Fig. 30. f9:**
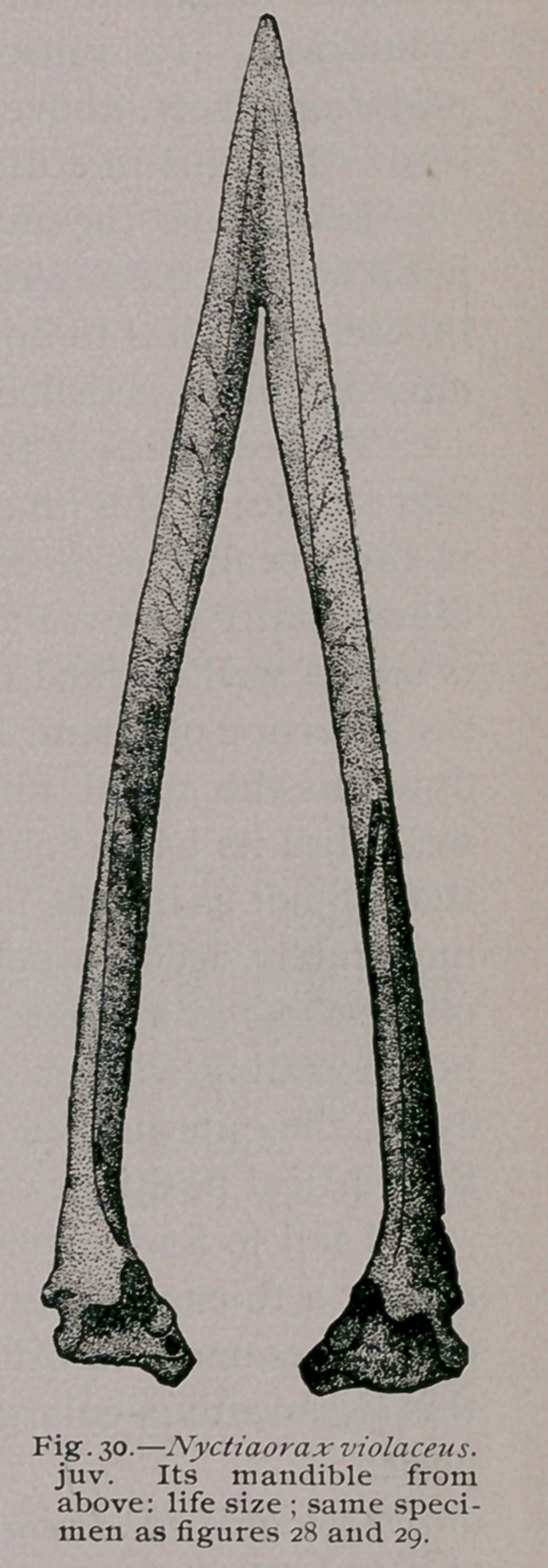


**Fig. 31. f10:**
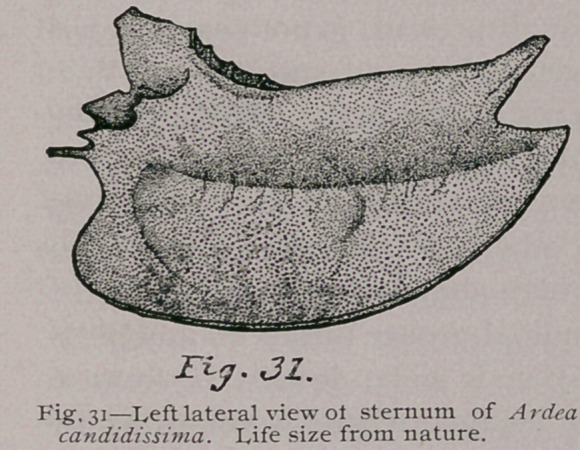


**Fig. 32. f11:**
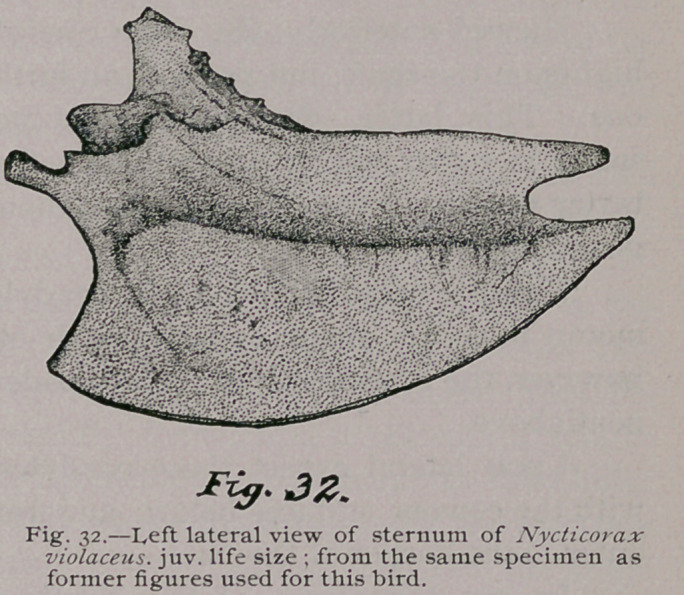


**Fig. 33. Fig. 34. f12:**
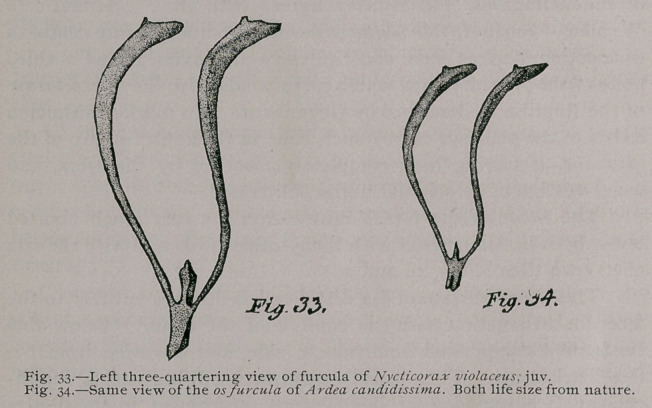


**Fig. 35. Fig. 36. Fig. 37. f13:**